# Effect of Short-Term Metro-Rhythmic Stimulations on Gait Variability

**DOI:** 10.3390/healthcare9020174

**Published:** 2021-02-06

**Authors:** Katarzyna Nowakowska-Lipiec, Robert Michnik, Sandra Niedzwiedź, Anna Mańka, Patrycja Twardawa, Bruce Turner, Patrycja Romaniszyn-Kania, Aneta Danecka, Andrzej W. Mitas

**Affiliations:** 1 Department of Biomechatronics, Faculty of Biomedical Engineering, Silesian University of Technology, 41-800 Zabrze, Poland; Robert.Michnik@polsl.pl (R.M.); Aneta.Danecka@polsl.pl (A.D.); 2 Students Scientific Circle “Biokreatywni”, Department of Biomechatronics, Faculty of Biomedical Engineering, Silesian University of Technology, 41-800 Zabrze, Poland; s.niedzwiedz@interia.eu; 3 Department of Informatics and Medical Devices, Faculty of Biomedical Engineering, Silesian University of Technology, 41-800 Zabrze, Poland; Anna.Manka@polsl.pl (A.M.); patrycja.twardawa@gmail.com (P.T.); Patrycja.Romaniszyn-Kania@polsl.pl (P.R.-K.); Andrzej.Mitas@polsl.pl (A.W.M.); 4 dBs Music, HE Facility, 17 St Thomas Street, Bristol BS1 6JS, UK; bruce.turner@dbsmusic.co.uk

**Keywords:** biomechanics, gait, gait frequency, rhythmic auditory stimulation, treadmill

## Abstract

The aim of the study was to define the effect of different short-term metro-rhythmic stimulations on the time and spatial parameters of gait. The secondary goal was to test whether prior instructions on how to respond to stimulations played a significant role in the stimulation by sound stimuli. Experimental tests of gait were conducted on a group of 36 healthy participants: group 1—subjects who were not informed how to react after hearing sound stimuli, group 2—subjects who received a clear instruction before the test to adjust the frequency of taking steps to the rhythm of the music. The gait research was carried out on a Zebris FDM-S (zebris Medical Gmbh, Isny, Germany) treadmill for various sound stimuli (arrhythmic stimulus, rhythmic stimuli at different rate). It was shown that a short-term influence of metro-rhythmic stimulations changes the time and spatial parameters of gait, i.e., gait frequency, length and duration of the gait cycle. The greatest impact on the modification of the time–space parameters of walking is exerted by rhythmic stimuli at a pace different from the frequency of gait at a preferred velocity. Providing information on how to respond to sounds heard may be important in gait therapy with RAS (rhythmic auditory stimulation).

## 1. Introduction

The human body’s nervous system is an essential component responsible for balance and locomotion, allowing for the gait’s complex motor activity. According to the ICD-10 International Classification of Diseases, mobility impairment is included among symptoms and features of diseases that also affect the musculoskeletal system [1]. Statistics presented by the World Health Organization show that about 10% of the population between the ages of 60 and 69, and more than 60% over 80, suffer from a gait disorder [1]. These data present how locomotor disorders are a global problem, most often occurring as a consequence of diseases and conditions such as Alzheimer’s disease (AD), Parkinson’s disease (PD), stroke, muscular dystrophy or multiple sclerosis (MS) [2]. In most cases, unfortunately, gait or balance pathologies cannot be fully cured, so it is essential to build on existing methods and look for new ones to restore motor skills to the highest possible level.

Rebuilding the locomotor function is a long-term and resource-intensive process. The critical stage is to restore the patient’s self-balance while standing, and the most commonly used therapeutic technique for this is proprioceptive neuromuscular facilitation (PNF) [3]. There is also an increasing trend to use specialized rehabilitation equipment to improve patients [4].

In recent years, there has been new evidence indicating that therapy with the use of sounds and rhythm reduces neurological dysfunctions and slows down the deterioration of cognitive functions [5,6]. However, in the attempt to combine traditional rehabilitation with sensomotor stimulation based on sound stimuli, one should still look for and search for new relationships between the stimulant used and the effects obtained.

The use of rhythmic stimuli in gait rehabilitation was defined as a rhythmic auditory stimulation (RAS) [7] by Thaut et al. Rhythmic auditory stimulation initiates the proper execution of movement in real time and is used as a stimuli in therapy. The method using sound stimulation is characterized by acoustic stimuli adjusted to the basic frequency of the patient’s gait or the use of music patterns with a rhythm greater or smaller than the basic frequency of gait [7]. Every RAS therapy should be adjusted to the needs of the patient and their disability in order to achieve improvement of certain movement parameters, which should be as close as possible to the values obtained in people without any movement disorders [8]. The kinds of stimuli used and their characteristics may have a different influence on the movement [9]. The type of music, especially time patterns, may affect the change of gait velocity and stride length [10]. Another element that appears indirectly when using musical cues are the emotions they evoke [11]. Pleasant musical emotions are associated with acceleration of gait and lengthening of stride, while music that evokes unpleasant emotional states causes the opposite. The influence of these emotions also depends on the familiarity with music.

So far, the simulations have involved the use of the sound of a metronome [9,12,13,14,15,16,17,18], natural sounds of steps (referred to as ecological RAS) [12], well-known music compositions [9] as well as clapping and clicking [19].

Conducted research confirms the effectiveness of acoustic stimuli in the minimization of movement deficits in patients after stroke incidents, in Parkinson’s disease or in people suffering from multiple sclerosis [12,13,14,15,16,19,20,21,22,23,24,25,26,27]. In this research evaluating the effectiveness of RAS, treadmill walking was most often analyzed [9,13,26] due to the possibility of controlling the patient’s gait velocity and a simple method of adjusting the stimulus to the walking pace. The study by Murgia et al. compared the effects of the rehabilitation of 32 patients with PD integrated either with ecological or artificial RAS [12]. The results suggest that ecological RAS is equally as effective as compared to artificial RAS. A team of researchers led by Yoon aimed to determine whether inclined treadmill walking combined with RAS improves gait and balance ability in post-stroke patients [13]. Shin et al., in their study, examined the effect of gait training with RAS on kinematic gait parameters in patients with hemiplegia [14]. Another study by Wright et al. reviewed whether a metro-rhythmic cue during gait reduces excessive variability in gait parameters after stroke [15]. In patients with the same disease entity, the effect of RAS use on the impact of symmetrization of lower limb loads was studied [28]. Ko et al. determined the relationship between RAS rate and gait quality in post-stroke patients [19]. The effectiveness of using metro-rhythmic stimulation on treadmill gait of post-stroke patients to improve coordination was also evaluated in 2007 by a team led by Roerdink [26]. A study of the effect of rhythmic auditory stimulation on gait kinematic parameters was also performed in 18 patients with multiple sclerosis as an effective rehabilitation method [16]. Another study showed that RAS could also affect quantitative gait parameters in MS patients [22].

Patients with PD experience moments of freezing of gait (FoG). A study by authors Arias and Cudeiro evaluated RAS’s effects in patients with FoG episodes at the end of dose periods [20]. Researchers from France looked at determining whether metro-rhythmic stimulation contributes to improving the preparation or execution of movement initiation in PD patients’ FoG episodes [23]. Another team from Israel investigated the hypothesis that rehabilitation that uses RAS can alleviate FoG symptoms [24]. Patients with PD also have difficulty learning new motor skills, so Rochester et al. [25] examined the effect of using external rhythmic stimulation to acquire new motor skills in patients with Parkinson’s disease. Willems et al. evaluated the effect of rhythmic auditory stimulation on gait in PD patients with and without FoG [27]. Lei et al. studied cognitive effects of rhythmic auditory stimulation in Parkinson’s disease and they indicate that RAS facilitates attentional processing in PD patients [29]. However, there is no effect of auditory–motor synchronization on temporal predictability in PD patients. The latest research by Nwebube et al. (2021) indicates that rhythmic auditory music stimulation increases task distraction during exercise among cardiac rehabilitation patients [30].

The changes of time and spatial parameters of gait were also noted in healthy people [9,17,18,31]. Wittwer et al., in their work, determined the effects of both rhythmic music and metronome on gait variability in older, healthy individuals [9]. Other studies have focused on analyzing gait dynamics with metro-rhythmic beats with forced parameters—speed or cadence [17]. Schreiber et al. looked at comparing gait characteristics in healthy individuals at preferred and reduced gait speed, with and without RAS application [18]. The ability to modify gait using rhythms depends on auditory–motor coordination, and these skills were tested on 20 healthy older adults in the article by Roerding et al. [31].

In the scientific research evaluating the effectiveness of RAS, treadmill walking was most often analyzed [9,13,17,26] due to the possibility of controlling the patient’s gait velocity and a simple method of adjusting the stimulus to the walking pace. There are also studies evaluating a short-term impact of RAS on locomotive functions. Such works present one-day tests attempting to determine the speed of adjusting the gait to the music pattern [19,20,27,31]. There are also studies that investigate the impact of long-term stimulation with metro-rhythmic stimuli [12,14,32].

Even though the scientific literature features many publications confirming the effectiveness of the application of acoustic stimuli to the therapy of gait, there is a lack of studies investigating various types of composed music pieces. Usually, a metronome [9,12,13,14,15,16,17,18] or short sound stimuli [12,19] were used in the studies of gait with RAS. In a long-term therapy with RAS, the nature and type of sound heard may have a significant meaning. Moreover, in view of the music characteristic and greater accumulation of information in it, a music stimulus has a greater influence on gait than a metronome. Additional pieces of information, such as melody, harmony and rhythm between main beats, allow the optimization of the motion path [9].

Music, which is a permanent element of every person’s life, affects the feelings and functioning by inducing subjective, behavioral and physiological changes [33]. Experiencing an intense reaction to music, which by stimulating proper autonomic systems contributes to the feeling of specific emotional states, is a frequent, repetitive and universal phenomenon for humans [33]. Undoubtedly, the kind of stimulation may have an influence on the patient’s mood [33,34,35,36].

It is also important to properly instruct the patient during synchronization. Giving proper cues on how to move while hearing a stimulus has different effects on patients with a strong and poor sense of rhythm [37,38]. It appears that providing short clues on how to react to sound stimuli while walking may have a significant meaning. The guidance given to the participants may help in adjusting both the spatial and temporal parameters of gait in a group of healthy adults, but there is still insufficient research that focuses on how verbal commands during rhythmic stimulation affect the gait.

The aim of the study was to define the effect of different short-term metro-rhythmic stimulations on the time and spatial parameters of gait. The secondary goal was to test whether prior instructions on how to respond to stimulations played a significant role in the stimulation by sound stimuli.

## 2. Materials and Methods

### 2.1. Participants

Students and employees of the public university where the research was conducted were invited to participate in the research. An informative campaign was carried out (verbally and e-mail), explaining the purpose of the research, the way it was conducted, the preparation for the study (footwear and sportswear), as well as the inclusion criteria. The inclusion criteria for the studies were as follows: adult people aged between 18 and 40 years old, with no locomotive function disorders, no previous surgeries in the area of the spine or lower limbs, no injuries within the last 3 months. The respondents reported by e-mail. A total of 38 people applied for the research. At the beginning of the study, each participant was interviewed to check whether the inclusion criteria for the study were met. Based on the interviews conducted, 36 out of 38 reported participants were qualified for further research. The cycle of studies was carried out in the period from June 2019 to January 2020.

All the test participants gave their informed consent to take part in the tests. The research was conducted according to the ethical principles stated in the Helsinki Declaration, and the protocol was approved by the Bioethics Committee of the Jerzy Kukuczka Academy of Physical Education in Katowice (No. 3/2019).

The test participants were divided into two equal groups:

- G1—group 1—subjects who were not informed about the aim of the test and the necessity of the adjustment of gait to the sounds heard;
- G2—group 2—subjects informed before the test about its aim and asked to adjust the frequency of taking steps to the sounds heard.

The characteristics of the examined subjects are presented in Table 1.

### 2.2. Measurement Protocol

**Figure 1:**
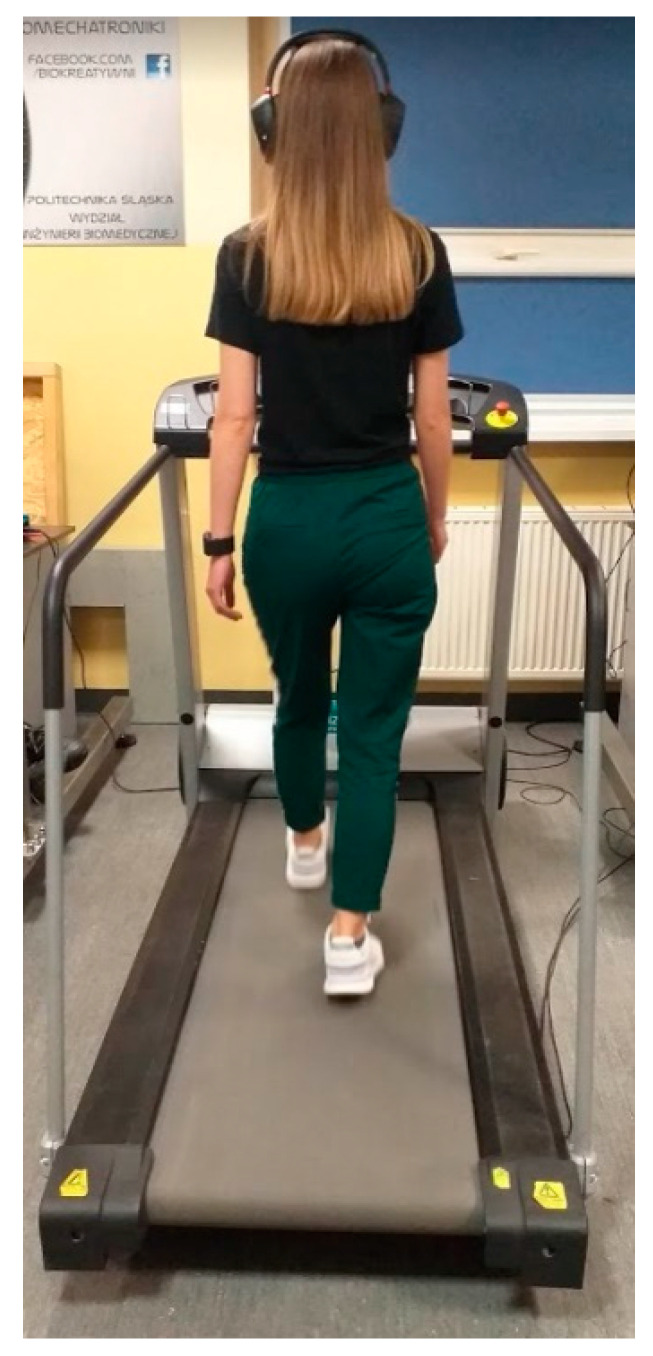
A participant during walking with the impact of acoustic stimuli.

The assessment of locomotor functions was performed during gait on a ZEBRIS FDM-T treadmill (ZebrisMedical GmbH, Isny, Germany). The WinFDM-T software was used to register the results, the sound stimuli were transmitted via wireless headphones Sound BlasterTactic 3D Rage Wireless V2.0 (Creative Technology Ltd., Singapore). The examined subjects walked on the treadmill in sports shoes (Figure 1). At the first stage, each test participant individually adjusted their preferable gait velocity when walking on the treadmill (vp-preferable gait velocity). The procedure for determining the preferred speed of treadmill walking was as follows: participants began to walk on a treadmill at a speed of 3 km/h, which was then increased by 0.1 km/h until the subject reported that the speed was preferred. Afterwards, the then-current speed was increased by 1.5 km/h and, next, decreased repeatedly by 0.1 km/h to return to the speed regarded as preferable. After each change of speed, the operator asked whether the then-present speed of gait was preferable. In the case of uncertainty, the procedure was repeated [39]. Each test participant, after establishing the preferred walking speed, walked on the treadmill for about 2 min to get used to it.

After establishing a preferred speed of gait, each test participant walked on the treadmill for about 2 min in order to get used to it. Thereafter, a 60-s gait at a preferred velocity (vp) was registered (GP—reference test) and the results of this test were used to determine the average walking frequency at a preferred velocity. Subsequent measurements included the gait with metro-rhythmic stimuli during which the velocity set on the treadmill corresponded to a preferred walking speed. Each measurement lasted 120 s. For the first 30 s no sound was played, and the examined person walked in accordance with their own rhythm of steps. Next, one of the sound stimuli was played and the gait with stimulation lasted 90 s [39]. The applied metro-rhythmic stimuli were prepared by an educated musician.

The tests were performed in relation to four types of stimulation:

- GA (arrhythmic stimulus played at a rate of 120 BPM, time 4/4, ambient style)—the lack of accents in the stimulus and gradual transition between individual tones. Music was supposed to provide relaxation, assumedly leading to the symmetrization and “tranquilizing” of gait;
- GR100 (rhythmic stimulus played at a rate corresponding to the frequency of gait and determined during tests of preferable speed, 4/4 time, motivating music). The stimulation was of a motivating nature, characteristic of music played during sports training (e.g., aerobics);
- GR110 (rhythmic stimulus as above, played at a rate corresponding to gait frequency increased by 10%, the tempo was determined during the tests of gait at a preferable speed);
- GR200 (rhythmic stimulus as above, played at a rate corresponding to doubled gait frequency, the tempo was determined during the tests of gait at a preferable speed) [39].

### 2.3. Results Analysis Procedure

The assessment involved the susceptibility of healthy test participants to metro-rhythmic stimuli. The following gait parameters were analyzed: the length of the gait cycle, the time of the gait cycle, the width of stride and the frequency of taking steps. The coefficient of the variation of the analyzed gait parameters was also assessed.

The analyzed parameters were determined for 45-s time periods:

- For gait without metro-rhythmic stimuli (GP) from 15 to 60 s of registered gait;
- For gait with metro-rhythmic stimuli (GA, GR100, GR110, GR200) from 60 to 105 s, which means the analysis of the fragment beginning 30 s after the appearance of the sound stimuli [39].

The analysis of the results was carried out with a division into two groups (G1, G2).

An analysis of the variability of the analyzed parameters in subsequent gait tests was performed. The coefficient of variation (CV) was determined for all parameters for each of the examined subjects and for all subsequent tests. The percentage differences in the coefficient of variation of the analyzed parameters were determined between the tests with metro-rhythmic stimuli and the reference test (without stimuli).

The results were analyzed statistically. The quantitative variables of the analyzed gait parameters were described using the mean value, standard deviation as well as the minimum and maximum value. The normality of the distribution of the analyzed variables was verified using the Shapiro–Wilk test. The presence of differences between the analyzed parameters of gait for two groups (G1, G2) was verified by performing the Student’s *t*-test for independent samples or the Mann–Whitney U test, depending on the normality of the distribution of analyzed variables. In order to determine statistically significant differences between the analyzed gait parameters in the GP reference study and subsequent tests with sound stimuli, the Student’s T-test for dependent samples or the Wilcoxon test was used, depending on the normality of the distribution of analyzed variables. The level of significance adopted in the statistical analyses was *p* = 0.05. Calculations were performed using a Statistica 13.1 software programme (Round Rock, TX, USA).

## 3. Results

The results of time and spatial parameters of gait obtained in tests GP, GA, GR100, GR110 and GR200 for groups G1 and G2 are presented in Table 2.

The statistical tests showed that the analyzed time and spatial parameters did not significantly differ between groups G1 and G2 for the gait with the preferred velocity without sound stimuli (GP). There were also no differences between tests GR100 and GR200 conducted in both groups. For the gait with arrhythmic sound (GA), statistical differences between the groups were obtained for the length of the gait cycle. In the test involving rhythmic sounds with the pace 10% faster than the basic walking frequency (GR100), statistically significant differences between groups G1 and G2 were obtained for the frequency of taking steps and the time of the gait cycle.

The Student’s t-test for dependent samples or the Wilcoxon test were used to determine statistically significant differences between the GP test results and the following tests with metro-rhythmic stimuli GA, GR100, GR110 and GR200. The results are presented in Table 3.

**Figure 2:**
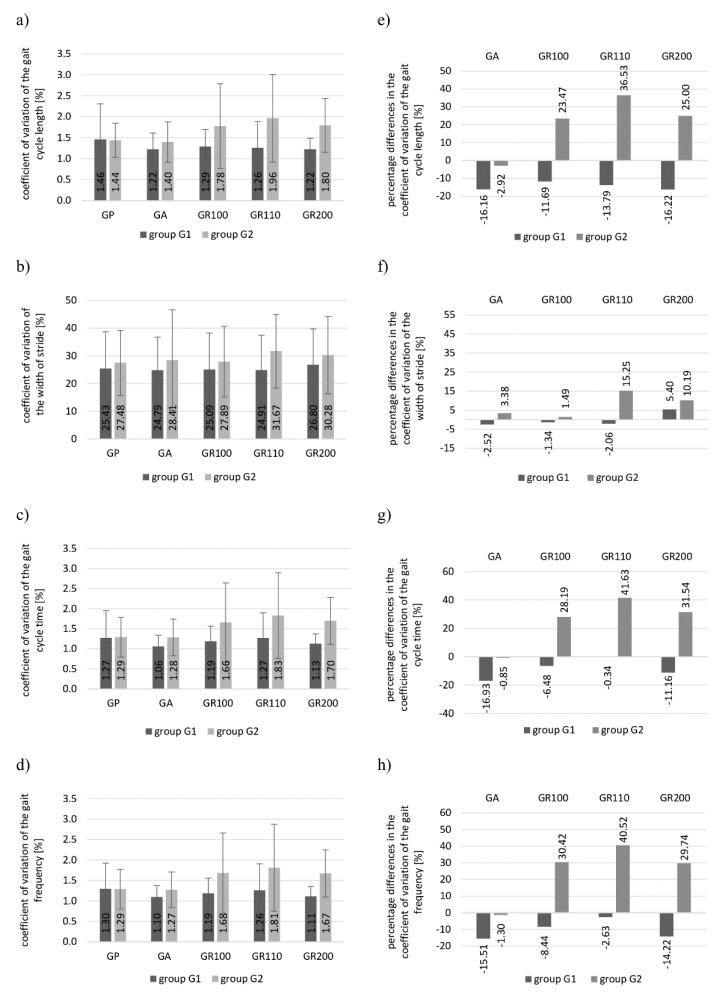
Coefficient of variation and percentage differences in the coefficient of variation for groups G1 and G2 obtained in particular tests for the analyzed parameters of gait: (**a**,**e**) length of the gait cycle; (**b**,**f**) stride width; (**c**,**g**) time of the gait cycle; (**d**,**h**) frequency of taking steps. (CV—coefficient of variation, G1—group 1, G2—group 2, RAS—rhythmic auditory stimulation, GP—reference gait test without auditory stimuli, GA—gait test with arrhythmic stimulus, GR100—gait test with rhythmic stimulus played at a rate corresponding to the frequency of gait, GR110—gait test with rhythmic stimulus played at a rate corresponding to gait frequency increased by 10%, GR200—gait test with rhythmic stimulus played at a rate corresponding to doubled gait frequency).

Figure 2a–d show the mean values and the standard deviation of the gait variation coefficient in groups 1 and 2 for the analyzed parameters and subsequent gait tests. Figure 2e,f present the percentage differences in the coefficient of the variation of the analyzed parameters between the tests with metro-rhythmic beats and the reference test.

## 4. Discussion

The studies conducted in accordance with the proposed procedure and the analysis of the obtained results enabled the determination of the effect of the short-term impact of metro-rhythmic stimuli on the change of time and spatial parameters of gait in healthy subjects. The study also made an attempt to determine whether providing prior information on how to react to the stimuli is an important element of the stimulation by the sound stimuli.

### 4.1. The Effect of Short-Time RAS Influence on the Change of Time and Spatial Parameters of Gait

The results of the conducted statistical tests showed that the time and spatial parameters of gait for the GP reference test did not differ between groups G1 and G2. In group G2, the length of the gait cycle was greater (on average by about 7 cm), as was the time of the gait cycle (by about 0.01 s on average). The frequency of taking steps was lower (by about 2 steps/s) and the width of the step was shorter (by about 3.5 cm). For the gait with arrhythmic sound, the intergroup differences were noted only for the length of the gait cycle (Table 2). The analysis of the mean values obtained in both groups in the GA test showed an increase in the length of the gait cycle (in group G1 by about 1.5 cm on average, in group G2 by almost 4 cm) and a decrease in the frequency of taking steps (in group G1 by approximately 1.5 step/min on average and in group G2 by 2.47 step/min). It can be concluded that this type of music provided relaxation and calmed down the pace of walking.

Similarly, no intergroup differences were noted for the gait with stimulation by rhythmic sounds of pace twice as high and equal to the frequency of the steps taken (tests GR100, GR200). In the study with the use of rhythmic sounds at a pace of 10% faster than the basic gait frequency (GR110), statistically significant differences between groups G1 and G2 were obtained for the frequency of taking steps and the cycle of gait. This may suggest that providing information on how to react to the sounds heard may have importance in the gait therapy with RAS. The analysis of the mean values of the parameters in group G2 showed an increase in the frequency of steps taken (by about 5 steps/min, which is approximately 5% regarding the frequency from the GP test, thus approaching the pace of the presented stimulus) and the shortening of the time of the gait cycle (on average by 0.05 s). Opposite dependences were noted in the G1 group—a reduction in the frequency of taking steps (on average by 2.58 steps/min) and an extension of the gait cycle (on average by 0.03 s, Table 2.) In both groups, the differences of the analyzed parameters between the GP and GR110 tests are statistically significant (Table 3).

It was proven that in group G1 the length and time of the gait cycle as well as the frequency of taking steps showed significant differences between the GP reference test and all tests involving sound stimulations (GR100, GR110, GR200). In group G2, differences for these parameters were shown between the values obtained in GP and GA together with GR110. In the case of subjects who were provided with prior instructions on how to react to the sounds heard, these parameters did not significantly differ in the GR100 and GR200 tests with reference to the GP test. Moreover, it was noticed that sound stimuli during gait did not influence the change of the stride width. Statistically significant differences in the stride width in relation to the reference test were noted just in one case: in group G2 for test GR110 (the mean difference was 1.08 cm).

### 4.2. The Variability of Gait in Healthy Subjects during Stimulation with Metro-Rhythmic Stimuli

Interesting dependences can be found by analyzing the values of the variation coefficient of the gait parameters and its percentage differences between the values obtained in the tests with metro-rhythmic stimuli and the reference test (Figure 2).

The coefficient of variation of all analyzed parameters (gait cycle length, stride width, gait cycle time, step frequency) in the GP test was on a comparable level in both groups. The lowest variability for all parameters was noted for the GA test, i.e., for the gait with an arrhythmic stimulus, for which when the music played had a relaxing function, it calmed the pace of gait.

The gait tests with rhythmic stimuli (GR100, GR110, GR200) showed a significantly higher CV for all parameters in group 2. It is also confirmed by the percentage differences in the variation coefficient of parameters between the tests with metro-rhythmic stimuli and the tests without stimuli.

In the investigations with rhythmic stimuli, GR100, GR110 and GR200, the percentage

CV differences of the gait frequency in group 2 were 30%, 41% and 30%, respectively, while in group 1 they did not exceed 15%. The greatest change of the parameters of gait in group 2 was noted for the GR110 test, probably due to the fact that the pace of metro-rhythmic stimuli did not correspond to the gait frequency for the set preferred walking speed on the treadmill. The above-mentioned fact caused the examined subjects to attempt to adjust their gait to it. The pace of stimuli was significantly higher in the GR200 test. Following the investigations, some subjects from group G2 declared that they tried to walk according to every second bar, while others felt confused. Therefore, after some time they abandoned their attempts to adjust the frequency of walking to the sounds they heard. The percentage CV differences in length and time of the gait cycle in group 2 in the tests with rhythmic stimuli were on a similar level. The greatest differences were noted in the GR110 test: for the length of the gait cycle, it amounted to 37%, and for the time of the gait cycle to 42%. In the case of the GR100 and GR200 tests, they amounted to 24% and 25%, respectively, for the length of the gait cycle as well as 28% and 32% for the time of the gait cycle. The greatest percentage CV differences for the stride width in test 2 were also noted in tests GR110 (15%) and GR200 (10%).

It is interesting that the highest CV in group G1 for all analyzed gait parameters was noted in the GP test (the only exception being the stride width in the GR200 test). As follows from the above, the gait of the subjects who did not receive information how to react to the sounds heard was less varied in the tests with auditory stimulation (smaller standard deviations of the parameters during walking). The percentage CV differences for the analyzed parameters between the tests with stimuli and the GP did not exceed 20%. In the GR110 test, the percentage CV differences with reference to the GP did not exceed 3% for the time of the gait cycle and the frequency of taking steps.

Summarizing, a much higher variability of the analyzed parameters of gait was noted in group 2, i.e., in the subjects who received clear prior instructions to try to walk to the rhythm of the music after hearing the metro-rhythmic stimuli. The examined subjects undertook such attempts, therefore in the investigations with rhythmic stimuli, greater deviations of the analyzed gait parameters were noted than in the GP test. The gait of the subjects from group 1 in the tests with sound stimuli was less varied than in the GP test. By analyzing the values of frequency in the subsequent studies, it can be concluded that the subjects from group 1 did not attempt to adjust the frequency of taking steps to the pace of sounds. An exceedingly important aspect of the gait with RAS is to provide the examined subjects with prior clues on how to react to the sounds heard. On the basis of the conducted analyses, it can also be concluded that the modification of the time and spatial parameters of gait is most significantly influenced by the rhythmic stimuli with a pace different from the frequency of the gait with a preferred speed. However, their pace should not differ significantly from the frequency of the steps taken.

### 4.3. Own Results Compared to the Research Conducted by Other Authors and Their Importance in Rehabilitation

The results obtained in this study indicate that the short-term metro-rhythmic stimuli affect the change of time and spatial parameters of gait, specifically on the frequency of gait, length and time of the gait cycle. The analysis of mean values of the parameters in group G2 showed an increase in the frequency of steps taken and the shortening of the time and length of the gait cycle. However, statistically significant differences between the results obtained in the GP test and following tests with metro-rhythmic stimuli in group 2 were noticed only for tests with GR110 stimuli. We conclude that a frequency of stimulus of 110% of the preferred walking cadence is the most effective stimulus. Interestingly, opposite dependences were noted in the G1 group, i.e., a reduction in the frequency of taking steps and an extension of the gait cycle in gait with RAS. Subjects from group 1 did not receive clear prior instructions to try to walk to the rhythm of the music after hearing the metro-rhythmic stimuli. It can be concluded that providing instructions on how to respond to stimulations played a significant role in short-term stimulation by sound stimuli.

The scientific research conducted by other authors also showed that the rhythmic auditory stimulation influences the change of time and spatial parameters in healthy persons [9,17,18,31] and in patients who suffer from neurological disorders [12,13,14,15,16,19,21,23,24]. The application of sound stimuli had a positive effect on the change of the gait frequency in the tests in which the rhythm of the sound used was equal to or greater than the cadence [9,12,13,16,19,22,26,32], which is also confirmed by our studies. In the studies conducted so far, the frequency of the given signals was altered within the range of ±22.5% of the basic value of the examined subject’s gait frequency. The literature features also the studies analyzing the effect of rhythmic auditory stimulation on the change of the length of the gait cycle [9,12,14,16,18,19,22,26] and the time of the gait cycle [9,14,16,19,26]. In the research by Wittwer et al. [9], there was an increase in the length of the gait cycle under the influence of sound stimuli by 2.31% [9], whereas the sound of the metronome increased this value by 0.77% [40]. At the same time, there are studies in which the length of the gait cycle with the use of RAS for the preferred speed fell by 2.47% [25]. These values are close to the results presented in this study: in group G1 for the GR110 test, an increase in the length of the gait cycle by 2.56% was noted; whereas in group G2, a decrease by 4.44%. Both in the literature [9,14,16,19,26] and in our own studies (for group G2), the shortening of the time of the gait cycle was indicated in the tests in which an increased pace of sounds was applied.

Erra et al. [41] indicated that RAS instantly improves parkinsonian gait and that a frequency of 110% of the preferred walking cadence is the most effective stimulus, which is also confirmed by our research. Erra et al., like other scientists, point out that RAS improves spatio–temporal parameters and gait phase distribution, but they additionally indicate that RAS leaves joint kinematics unaltered [41]. In a recent study by Thaut et al., they indicate that RAS training significantly reduced the number of falls in Parkinson’s disease and modified key gait parameters, such as velocity and stride length [42].

Styns et al. [43] showed that the subjects walk faster to music rather than to a metronome. The examined subjects were able to synchronize their gait with these kind of stimuli, but they did it in the most effective way when the pace of the stimuli was close to 120 BPM [43]. Wittwer et al. [9] defined the influence of the music and metronome stimuli on the gait parameters in healthy elderly persons. At the beginning of the test, the variability of the time and spatial parameters was low and did not change under the influence of the two stimuli (metronome, music). However, the subjects walked faster while listening to music [9]. The results of the studies suggest that the assessment of the influence of RAS on gait should take into consideration the type of the stimuli and its frequency.

Other analyses, conducted by Nowakowska-Lipiec et al. [38] on a smaller group of healthy subjects, showed that the presented short-term rhythmic sound stimuli with the pace equal to taking steps with a preferred gait velocity had an influence on the symmetrization of the time of taking steps. However, no clear short-time influence of RAS on the change of the symmetry of the stride width was noted [38]. In our research, there were also no significant changes in step width under the short-time influence of RAS.

The analysis of the coefficient of variation in our research showed that much higher variability of the analyzed parameters of gait was noted in group 2, i.e., in the subjects who received clear prior instructions to try to walk to the rhythm of the music after hearing the metro-rhythmic stimuli. Higher values of the standard deviation of parameters were observed for them in the tests with metro-rhythmic stimuli than in the GP test. The examined persons attempted to walk to the rhythm of the sounds heard, which may result in higher CV values. In this study, the long-term effect of stimulation with metro-rhythmic stimuli on gait variability was not investigated. However, studies by del Olmo and Cudeiro et al. [44] and Hausdorff et al. [45] indicate that long-term RAS interactions may lead to a lower variability of gait parameters. Del Olmo and Cudeiro et al. studied the influence of RAS on sequential movements in patients with Parkinson’s disease (PD). It was shown that after 4 weeks of rehabilitation with RAS, the patients’ gait coefficients of variability improved significantly for the preferred gait [44]. The results of the research by Hausdorff et al. also demonstrate that RAS enables more automatic movement and reduces stride-to-stride variability in patients with PD. The authors indicate that these improvements are not simply a by-product of changes in speed or stride length [45].

It should be noted that, so far, no studies have been conducted in which the subjects would be stimulated by composed arrhythmic pieces of music and rhythmic ones with a variable pace depending on the individual cadence of gait of the subject. It should also be noted that the sound of the metronome in longer rehabilitation of gait can be an irritating sound. So far, the scientific literature has not raised the issue of the role of the provision of prior instructions/clues how to respond to sound stimuli during the gait with metro-rhythmic stimuli.

An extremely important aspect, which should be included in further research, is the fact that the results of gait with RAS may be dependent on an innate ability to extract rhythm from the outside world and on a mechanism called “entrainment” that provides the synchronization of movements to the rhythm [41,46,47]. The neuronal basis of entrainment is based on the well-documented connections between the auditory and motor systems [41,48,49,50]. It is not known how different kinds of RAS influence gait entrainment.

To summarize, RAS is a useful, low cost, rehabilitation tool. According to Erra et al. [41], increasing walking speed and reducing stride time could have a great impact on the reduction of bradykinesia symptoms. This can be very helpful for disabled people in performing everyday movements in daily life activities. The future of using RAS in daily rehabilitation is dedicated to unobtrusive devices to permit safe RAS integration in patients’ daily life, adapting the auditory stimulus to the natural walking pattern. However, this requires further research to verify whether RAS-related gait improvements persist in a daily life setting.

### 4.4. Limitation of This Study and Directions of Further Research

Some limitations of the presented studies and analyses can be indicated. The studies were conducted on a treadmill, which could have an influence on the time and spatial parameters as well as kinematic gait patterns of the subjects. In many previous studies, the ground gait was compared to the gait on a treadmill, but the results were often conflicting and inconclusive [40,51,52,53]. In subsequent stages of studies, a similar experiment needs to be conducted for gait that is not forced by a treadmill. During such an experiment, measuring footwear insoles could be used. In addition, the tests with the use of metro-rhythmic stimuli in the form of composed music pieces were conducted exclusively on a group of healthy subjects. The studies need to be extended to the group of subjects with dysfunctions of locomotive functions. The impact of long-term metro-rhythmic stimuli on gait variability will be analyzed in the next phase of the studies. The investigations may also be extended to other types of sound stimuli. An interesting direction for further research would also be the determination of the impact of the provided stimulation on physiological parameters of the organisms of examined subjects.

## 5. Conclusions

The analysis of the obtained results enabled the formulation of the following conclusions:

- Short-term effect of metro-rhythmic stimuli during gait influences the change of time and spatial parameters of gait, i.e., on the frequency of gait, length and time of the gait cycle;
- When the music has a relaxation function, arrhythmic stimuli influence indirectly the mollification of the gait pace;
- Rhythmic stimuli with a pace different from the frequency of gait with a preferred speed have the strongest influence on the modification of the time and spatial parameters of gait, although their pace should not significantly differ from the frequency of the steps taken;
- Providing instructions on how to respond to stimulations played a significant role in the stimulation by sound stimuli—higher variability of the temporospatial parameters of gait was noted in group 2.

## Figures and Tables

**Table 1:** The characteristics of the examined subjects.

Variable	G1	G2	Difference (*p*-Value)	95% CI
Number of participants	18	18		
Sex (F/M)	14/4	12/6		
Age (mean ± SD) [years]	22 ± 2	25 ± 6	−3 (*p* = 0.03 *)	−6.08 to 0.08
Body mass (mean ± SD) [kg]	68.39 ± 16.94	67.22 ± 12.49	1.17 (*p* = 0.59)	−9.07 to 11.41
Body height (mean ± SD) [m]	1.72 ± 0.08	1.71 ± 0.07	0.01 (*p* = 0.65)	−0.04 to 0.07
Preferable gait speed (mean ± SD) [km/h]	3.98 ± 0.82	4.39 ± 0.44	−0.41 (*p* = 0.04 *)	−0.87 to 0.05

* *p* ≤ 0.05; G1—group 1, G2—group 2, F—female, M—male, SD—standard deviation.

**Table 2:** Results of time and spatial parameters of gait in tests GP, GA, GR100, GR110, GR200 for groups G1 and G2.

Gait Test	Parameter	G1		G2		G1 vs. G2
		Mean ± SD	Min–Max	Mean ± SD	Min–Max	*p*-Value
GP	length of the gait cycle [cm]	130.85 ± 9.62	109.07–147.23	137.84 ± 13.91	117.76–171.80	0.088
	width of stride [cm]	8.8 ± 3.18	3.15–16.26	8.43 ± 2.25	4.03–11.87	0.687
	time of the gait cycle [s]	1.09 ± 0.04	1.03–1.15	1.11 ± 0.06	1.00–1.22	0.218
	gait frequency [steps/min]	110.09 ± 3.82	104.01–116.07	108.22 ± 5.48	98.19–120.04	0.241
GA	length of the gait cycle [cm]	132.17 ± 8.82	113.82–148.34	141.59 ± 15.12	120.31–176.32	0.029 *
	width of stride [cm]	8.58 ± 3.02	3.18–16.38	7.95 ± 2.52	2.01–12.06	0.499
	time of the gait cycle [s]	1.11 ± 0.04	1.03–1.19	1.14 ± 0.06	1.03–1.25	0.071
	gait frequency [steps/min]	108.58 ± 4.4	101.26–116.31	105.48 ± 5.81	95.76–116.46	0.079
GR100	length of the gait cycle [cm]	132.51 ± 8.8	115.22–148.45	135.3 ± 14.28	115.93–169.13	0.485
	width of stride [cm]	8.69 ± 3.05	4.13–17.24	7.96 ± 2.56	3.61–13.8	0.446
	time of the gait cycle [s]	1.1 ± 0.05	1.04–1.2	1.09 ± 0.07	0.96–1.22	0.484
	gait frequency [steps/min]	108.81 ± 4.44	99.79–114.97	110.46 ± 7.13	98.76–125.81	0.411
GR110	length of the gait cycle [cm]	134.19 ± 9.59	114.95–149.56	131.73 ± 13.86	105.99–153.5	0.54
	width of stride [cm]	8.55 ± 2.87	3.55–15.93	7.35 ± 2.31	3.8–12.11	0.176
	time of the gait cycle [s]	1.12 ± 0.04	1.06–1.19	1.06 ± 0.07	0.97–1.28	0.002 *
	gait frequency [steps/min]	107.51 ± 4.11	100.64–113.77	113.55 ± 7.03	93.99–123.92	0.003 *
GR200	length of the gait cycle [cm]	133.83 ± 9.08	114.33–147.54	134.39 ± 13.1	117.36–158.92	0.882
	width of stride [cm]	8.83 ± 3.02	4.02–16.23	7.67 ± 2.76	3.49–13.25	0.240
	time of the gait cycle [s]	1.12 ± 0.04	1.06–1.19	1.09 ± 0.09	0.9–1.26	0.215
	gait frequency [steps/min]	107.71 ± 4.02	100.94–113.44	111.32 ± 9.63	95.54–133.92	0.152

* *p* ≤ 0.05; G1—group 1, G2—group 2, RAS—rhythmic auditory stimulation, GP—reference gait test without auditory stimuli, GA—gait test with arrhythmic stimulus, GR100—gait test with rhythmic stimulus played at a rate corresponding to the frequency of gait, GR110—gait test with rhythmic stimulus played at a rate corresponding to gait frequency increased by 10%, GR200—gait test with rhythmic stimulus played at a rate corresponding to doubled gait frequency.

**Table 3:** Results of the Student’s t-test or the Wilcoxon test determining statistically significant differences between the results obtained in the GP test and the following tests with metro-rhythmic stimuli: GA, GR100, GR110, GR200.

Gait Parameters	Type of Metro-Rhythmic Stimulation	GP vs. Gait with RAS	
		G1	G2
length of the gait cycle [cm]	GA	0.052	≤0.001 *
	GR100	0.004 *	0.057
	GR110	≤0.001 *	0.002 *
	GR200	≤0.001 *	0.181
width of stride [cm]	GA	0.246	0.052
	GR100	0.595	0.065
	GR110	0.253	0.003 *
	GR200	0.893	0.063
time of the gait cycle [s]	GA	0.001 *	0.001 *
	GR100	0.009 *	0.061
	GR110	≤0.001 *	0.003 *
	GR200	≤0.001 *	0.170
gait frequency [steps/min]	GA	0.001 *	≤0.001 *
	GR100	0.008 *	0.060
	GR110	≤0.001 *	0.001 *
	GR200	≤0.001 *	0.146

* *p* ≤ 0.05; G1—group 1, G2—group 2, RAS—rhythmic auditory stimulation, GP—reference gait test without auditory stimuli, GA—gait test with arrhythmic stimulus, GR100—gait test with rhythmic stimulus played at a rate corresponding to the frequency of gait, GR110—gait test with rhythmic stimulus played at a rate corresponding to gait frequency increased by 10%, GR200—gait test with rhythmic stimulus played at a rate corresponding to doubled gait frequency.

## Data Availability

The data presented in this study are available on request from the corresponding author. The data are not publicly available due to privacy and data protection protocols.

## References

[1] World Health Organization (WHO) International Statistical Classification of Diseases and Related Health Problems. https://www.who.int/classifications/icd/ICD10Volume2_en_2010.pdf.

[2] Jahn K., Zwergal A., Schniepp R. (2010). Gait disturbances in old age: Classification, diagnosis, and treatment from a neurological perspective. Dtsch. Arztebl. Int..

[3] Voss D.E., Ionta M.K., Myers B.J. (1985). Proprioceptive Neuromuscular Facilitation: Patterns and Techniques.

[4] Kania D., Romaniszyn P., Mańka A., Ledwoń D., Łysień A., Nawrat-Szołtysik A., Danch-Wierzchowska M., Michnik R., Mitas A., Myśliwiec A. (2020). Technology as a Support for Rehabilitation Patients After Stroke.

[5] Clements-Cortes A., Bartel L. (2018). Are we doing more than we know? possible mechanisms of response to music therapy. Front. Med..

[6] Fang R., Ye S., Huangfu J., Calimag D.P. (2017). Music therapy is a potential intervention for cognition of Alzheimer’s disease: A mini-review. Transl. Neurodegener..

[7] Thaut M.H., McIntosh G.C., Rice R.R., Miller R.A., Rathbun J., Brault J.M. (1996). Rhythmic auditory stimulation in gait training for Parkinson’s disease patients. Mov. Disord..

[8] Jochymczyk-Woźniak K., Nowakowska K., Michnik R., Gzik M., Kowalczykowski D., Tkacz E., Gzik M., Paszenda Z., Piętka E. (2019). Three-dimensional adults gait pattern—Reference data for healthy adults aged between 20 and 24. Proceedings of the Innovations in Biomedical Engineering.

[9] Wittwer J.E., Webster K.E., Hill K. (2013). Music and metronome cues produce different effects on gait spatiotemporal measures but not gait variability in healthy older adults. Gait Posture.

[10] Leman M., Moelants D., Varewyck M., Styns F., van Noorden L., Martens J.-P. (2013). Activating and relaxing music entrains the speed of beat synchronized walking. PLoS ONE.

[11] Ready E.A., McGarry L.M., Rinchon C., Holmes J.D., Grahn J.A. (2019). Beat perception ability and instructions to synchronize influence gait when walking to music-based auditory cues. Gait Posture.

[12] Murgia M., Pili R., Corona F., Sors F., Agostini T.A., Bernardis P., Casula C., Cossu G., Guicciardi M., Pau M. (2018). The use of footstep sounds as rhythmic auditory stimulation for gait rehabilitation in Parkinson’s disease: A randomized controlled trial. Front. Neurol..

[13] Yoon S.K., Kang S.H. (2016). Effects of inclined treadmill walking training with rhythmic auditory stimulation on balance and gait in stroke patients. J. Phys. Ther. Sci..

[14] Shin Y.-K., Chong H.J., Kim S.J., Cho S.-R. (2015). Effect of rhythmic auditory stimulation on hemiplegic gait patterns. Yonsei Med. J..

[15] Wright R.L., Bevins J.W., Pratt D., Sackley C.M., Wing A.M. (2016). Metronome cueing of walking reduces gait variability after a cerebellar stroke. Front. Neurol..

[16] Shahraki M., Sohrabi M., Taheri Torbati H.R., Nikkhah K., Naeimi Kia M. (2017). Effect of rhythmic auditory stimulation on gait kinematic parameters of patients with multiple sclerosis. J. Med. Life.

[17] Terrier P., Dériaz O. (2012). Persistent and anti-persistent pattern in stride-to-stride variability of treadmill walking: Influence of rhythmic auditory cueing. Hum. Mov. Sci..

[18] Schreiber C., Remacle A., Chantraine F., Kolanowski E., Moissenet F. (2016). Influence of a rhythmic auditory stimulation on asymptomatic gait. Gait Posture.

[19] Ko B.-W., Lee H.-Y., Song W.-K. (2016). Rhythmic auditory stimulation using a portable smart device: Short-term effects on gait in chronic hemiplegic stroke patients. J. Phys. Ther. Sci..

[20] Arias P., Cudeiro J. (2010). Effect of rhythmic auditory stimulation on gait in parkinsonian patients with and without freezing of gait. PLoS ONE.

[21] Bloem B.R., Hausdorff J.M., Visser J.E., Giladi N. (2004). Falls and freezing of gait in Parkinson’s disease: A review of two interconnected, episodic phenomena. Mov. Disord..

[22] Conklyn D., Stough D., Novak E., Paczak S., Chemali K., Bethoux F. (2010). A home-based walking program using rhythmic auditory stimulation improves gait performance in patients with multiple sclerosis: A pilot study. Neurorehabil. Neural Repair.

[23] Delval A., Moreau C., Bleuse S., Tard C., Ryckewaert G., Devos D., Defebvre L. (2014). Auditory cueing of gait initiation in Parkinson’s disease patients with freezing of gait. Clin. Neurophysiol..

[24] Plotnik M., Shema S., Dorfman M., Gazit E., Brozgol M., Giladi N., Hausdorff J.M. (2014). A motor learning-based intervention to ameliorate freezing of gait in subjects with Parkinson’s disease. J. Neurol..

[25] Rochester L., Baker K., Hetherington V., Jones D., Willems A.-M., Kwakkel G., Van Wegen E., Lim I., Nieuwboer A. (2010). Evidence for motor learning in Parkinson’s disease: Acquisition, automaticity and retention of cued gait performance after training with external rhythmical cues. Brain Res..

[26] Roerdink M., Lamoth C.J.C., Kwakkel G., van Wieringen P.C.W., Beek P.J. (2007). Gait coordination after stroke: Benefits of acoustically paced treadmill walking. Phys. Ther..

[27] Willems A.M., Nieuwboer A., Chavret F., Desloovere K., Dom R., Rochester L., Jones D., Kwakkel G., Van Wegen E. (2006). The use of rhythmic auditory cues to influence gait in patients with Parkinson’s disease, the differential effect for freezers and non-freezers, an explorative study. Disabil. Rehabil..

[28] Park K.S., Hass C.J., Fawver B., Lee H., Janelle C.M. (2019). Emotional states influence forward gait during music listening based on familiarity with music selections. Hum. Mov. Sci..

[29] Lei J., Conradi N., Abel C., Frisch S., Brodski-Guerniero A., Hildner M., Kell C.A., Kaiser J., Schmidt-Kassow M. (2019). Cognitive effects of rhythmic auditory stimulation in Parkinson’s disease: A P300 study. Brain Res..

[30] Nwebube C., Faulkner G.E., Thaut M.H., Bartel L.R., Stukel T.A., Redelmeier D.A., Marzolini S., Chen J.L., Goodman J.M., Oh P.I. (2021). Rhythmic auditory music stimulation increases task-distraction during exercise among cardiac rehabilitation patients: A secondary analysis of a randomized controlled trial. Psychol. Sport Exerc..

[31] Roerdink M., Bank P.J.M., Peper C.L.E., Beek P.J. (2011). Walking to the beat of different drums: Practical implications for the use of acoustic rhythms in gait rehabilitation. Gait Posture.

[32] Lee S., Lee K., Song C. (2018). Gait training with bilateral rhythmic auditory stimulation in stroke patients: A randomized controlled trial. Brain Sci..

[33] Juslin P.N., Västfjäll D. (2008). Emotional responses to music: The need to consider underlying mechanisms. Behav. Brain Sci..

[34] Balteş F.R., Avram J., Miclea M., Miu A.C. (2011). Emotions induced by operatic music: Psychophysiological effects of music, plot, and acting: A scientist’s tribute to Maria Callas. Brain Cogn..

[35] Abd-Elshafy S.K., Khalaf G.S., Abo-Kerisha M.Z., Ahmed N.T., Abd El-Aziz M.A., Mohamed M.A. (2015). Not all sounds have negative effects on children undergoing cardiac surgery. J. Cardiothorac. Vasc. Anesth..

[36] Nelson K., Adamek M., Kleiber C. (2017). Relaxation training and postoperative music therapy for adolescents undergoing spinal fusion surgery. Pain Manag. Nurs..

[37] Romaniszyn-Kania P., Kania D., Nowakowska K., Sobkowiak-Pilorz M., Turner B., Myśliwiec A., Michnik R., Mitas A. (2019). RAS in the aspect of symmetrization of lower limb loads. Proceedings of the International Conference on Information Technologies in Biomedicine, Kamień Śląski, Poland, 18–20 June 2019.

[38] Nowakowska-Lipiec K., Michnik R., Mańka A., Niedzwiedź S., Twardawa P., Romaniszyn P., Danecka A., Mitas A.W. Effect of various types of metro-rhythmic stimulations on the gait symmetry in healthy people. Proceedings of the 6th International Conference Engineering Mechanics.

[39] Michnik R., Nowakowska-Lipiec K., Mańka A., Niedzwiedź S., Twardawa P., Romaniszyn-Kania P., Turner B., Danecka A., Mitas A. (2021). Effect of various types of metro-rhythmic stimulations on the variability of gait frequency. Information Technologies in Medicine.

[40] Alton F., Baldey L., Caplan S., Morrissey M.C. (1998). A Kinematic comparison of overground and treadmill walking. Clin. Biomech. (Bristol).

[41] Erra C., Mileti I., Germanotta M., Petracca M., Imbimbo I., De Biase A., Rossi S., Ricciardi D., Pacilli A., Di Sipio E. (2019). Immediate effects of rhythmic auditory stimulation on gait kinematics in Parkinson’s disease on/off medication. Clin. Neurophysiol..

[42] Thaut M.H., Rice R.R., Braun Janzen T., Hurt-Thaut C.P., McIntosh G.C. (2019). Rhythmic auditory stimulation for reduction of falls in Parkinson’s disease: A randomized controlled study. Clin. Rehabil..

[43] Styns F., van Noorden L., Moelants D., Leman M. (2007). Walking on music. Hum. Mov. Sci..

[44] del Olmo M.F., Cudeiro J. (2005). Temporal variability of gait in Parkinson disease: Effects of a rehabilitation programme based on rhythmic sound cues. Parkinsonism Related Disord..

[45] Hausdorff J.M., Lowenthal J., Herman T., Gruendlinger L., Peretz C., Giladi N. (2007). Rhythmic auditory stimulation modulates gait variability in Parkinson’s disease. Eur. J. Neurosci..

[46] Nombela C., Hughes L.E., Owen A.M., Grahn J.A. (2013). Into the groove: Can rhythm influence Parkinson’s disease?. Neurosci. Biobehav. Rev..

[47] Moens B., Leman M. (2015). Alignment strategies for the entrainment of music and movement rhythms. Ann. N. Y. Acad. Sci..

[48] Bengtsson S.L., Ullén F., Ehrsson H.H., Hashimoto T., Kito T., Naito E., Forssberg H., Sadato N. (2009). Listening to rhythms activates motor and premotor cortices. Cortex.

[49] Grahn J.A., Brett M. (2009). Impairment of beat-based rhythm discrimination in Parkinson’s disease. Cortex.

[50] Bijsterbosch J.D., Lee K.-H., Hunter M.D., Tsoi D.T., Lankappa S., Wilkinson I.D., Barker A.T., Woodruff P.W.R. (2011). The role of the cerebellum in sub- and supraliminal error correction during sensorimotor synchronization: Evidence from FMRI and TMS. J. Cogn. Neurosci..

[51] Lee S.J., Hidler J. (2008). Biomechanics of overground vs. treadmill walking in healthy individuals. J. Appl. Physiol..

[52] Riley P.O., Paolini G., Della Croce U., Paylo K.W., Kerrigan D.C. (2007). A kinematic and kinetic comparison of overground and treadmill walking in healthy subjects. Gait Posture.

[53] Nymark J.R., Balmer S.J., Melis E.H., Lemaire E.D., Millar S. (2005). Electromyographic and kinematic nondisabled gait differences at extremely slow overground and treadmill walking speeds. J. Rehabil. Res. Dev..

